# Real-world effectiveness and safety of eculizumab in AQP4-IgG-positive neuromyelitis optica spectrum disorder

**DOI:** 10.1007/s00415-025-13608-w

**Published:** 2026-01-08

**Authors:** Emine Rabia Koc, Mehmet Fatih Yetkin, Furkan Saridas, Omer Faruk Turan, Serhan Sevim, Murat Terzi, Sedat Sen, Melih Tutuncu, Ugur Uygunoglu, Murat Kurtuncu, Tuncay Gunduz, Nermin Tepe, Sibel Canbaz-Kabay, Husnu Efendi, Sena Destan Bunul, Damla Cetinkaya-Tezer, İpek Gungor-Dogan, Serkan Demir, Burcu Altunrende, Nilufer Kale-Icen, Sami Omerhoca, Ozden Kamisli, Caner Fevzi Demir, Basak Karakurum-Yuksel, Cihat Uzunkopru, Yesim Beckmann, Vedat Cilingir, Kadriye Agan, Haluk Gumus, Merve Ercan, Belgin Kocer, Sanja Gluscevic, Edgar Carnero-Contentti, Adriana Casallas-Vanegas, Valentina Camera, Massimiliano Calabrese, Guven Ozkaya, Gulsen Akman-Demir, Cavit Boz, Ayse Altıntas, Aksel Siva

**Affiliations:** 1 Faculty of Medicine, Department of Neurology, Bursa Uludağ University, Bursa, Turkey; 2 Department of Neurology, Erciyes University, Kayseri, Turkey; 3 Department of Neurology, Mersin University, Mersin, Turkey; 4 Department of Neurology, Ondokuz Mayıs University, Samsun, Turkey; 5 Department of Neurology, İstanbul University-Cerrahpaşa, Istanbul, Turkey; 6 Department of Neurology, İstanbul University, Istanbul, Turkey; 7 Department of Neurology, Balıkesir University, Balıkesir, Turkey; 8 Department of Neurology, Dokuz Eylül University, Izmir, Turkey; 9 Department of Neurology, Kocaeli University, Kocaeli, Turkey; 10 Department of Neurology, Sancaktepe Şehit Prof. Dr. İlhan Varank TRH, Istanbul, Turkey; 11 Department of Neurology, Health Sciences University Taksim Training and Research Hospital, Istanbul, Turkey; 12 Department of Neurology, İstanbul Bagcılar TRH, Istanbul, Turkey; 13 Bursa Medical School, Department of Neurology, University of Health Sciences, Bursa, Turkey; 14 Department of Neurology, Fırat University, Elazig, Turkey; 15 Department of Neurology, Başkent University, Adana, Turkey; 16 Department of Neurology, İzmir Katip Çelebi University, Iznir, Turkey; 17 Department of Neurology, Van Yüzüncü Yıl University, Van, Turkey; 18 Department of Neurology, Marmara University, Istanbul, Turkey; 19 Department of Neurology, Selçuk University, Konya, Turkey; 20 Department of Neurology, Gazi University, Ankara, Turkey; 21 Department of Neurology, Clinical Centre of Montenegro, Podgorica, Montenegro; 22 Hospital AlemánCENRos, INECO Neurociencias Oroño, Rosario, Argentina; 23 Neuroimmunology Department. Cayre MS Centre, Facultad de Medicina, Universidad de La Sabana, Bogotá, Colombia; 24 Department of Neurology, University of Verona, Verona, Italy; 25 Department of Biostatistics, Faculty of Medicine, Uludag University, Bursa, Turkey; 26 Department of Neurology, İstanbul Bezmialem University, Istanbul, Turkey; 27 Department of Neurology, Karadeniz Technical University, Trabzon, Turkey; 28 Department of Neurology, Koc University, Istanbul, Turkey

**Keywords:** Neuromyelitis optica spectrum disorder, Eculizumab, Real-world study

## Abstract

**Objective:**

To evaluate the real-world effectiveness and safety of eculizumab in patients with AQP4-IgG–positive neuromyelitis optica spectrum disorder (NMOSD) and to identify predictors of disability outcomes.

**Methods:**

This multinational, retrospective cohort study analyzed data from 46 patients across 26 centers. The outcomes included the annualized relapse rate (ARR), relapse-free status, change in expanded disability status scale (EDSS) scores, and adverse events. To identify predictors of EDSS improvement or worsening, patients were stratified into subgroups (improved vs. stable/worsened) at each follow-up time point and compared based on demographic, clinical, and radiological variables.

**Results:**

This retrospective cohort study included 46 patients with AQP4-IgG-positive NMOSD from 26 centers, followed for a mean of 27.3 months. The mean ARR significantly decreased from 1.1 in the 2 years pre-treatment to 0.1 during eculizumab therapy. The relapse-free rate increased from 6.5% pre-treatment to 80.4%. Mean EDSS scores improved from 4.2 at baseline to 3.6 at 24 months. The presence of area postrema syndrome was associated with a favorable prognosis, while the presence of spinal attacks was associated with a poor prognosis at 12 months. Adverse events occurred in 7 patients (18.9%), leading to permanent discontinuation in only two.

**Conclusion:**

Eculizumab demonstrated robust real-world effectiveness in reducing relapse rates and stabilizing disability, with an acceptable safety profile. Clinical outcomes may be influenced by attack phenotype, underscoring the importance of early intervention.

## Introduction

Neuromyelitisoptica spectrum disorder (NMOSD) is a rare, severe autoimmune disease primarily affecting the central nervous system (CNS), predominantly characterized by recurrent episodes of optic neuritis (ON) and longitudinally extensive transverse myelitis (LETM) [1–3]. The majority of patients are seropositive for aquaporin-4 immunoglobulin G (AQP4-IgG), which plays a pathogenic role by inducing complement-mediated astrocyte injury. AQP4-IgG seropositivity serves as a diagnostic biomarker and therapeutic target, distinguishing NMOSD from other demyelinating diseases such as multiple sclerosis [4, 5]. In NMOSD, disability accumulation and progression are directly associated with relapse-related events. Preventing relapses is critical in NMOSD management, as each relapse can cause cumulative neurological damage and significant impairment in the patient’s quality of life [5–8].

Therapeutic options for AQP4-IgG-positive NMOSD have expanded significantly over the past decade. Historically, immunosuppressive agents, such as azathioprine and mycophenolate mofetil, have been commonly used to reduce relapse frequency. However, these treatments have variable efficacy and are associated with significant side effects. More recently, monoclonal antibodies such as rituximab, eculizumab (ECU), satralizumab, and inebilizumab have demonstrated superior efficacy in preventing relapses for AQP4-IgG-positive NMOSD. Among these therapeutics, ECU targets complement protein C5 and inhibits the formation of the membrane attack complex, which plays a pivotal role in astrocyte destruction initiated by AQP4-IgG [5, 7, 9–12].

By disrupting this step of the complement cascade, ECU effectively prevents relapses and associated disability. In the PREVENT study, ECU has demonstrated a significant reduction in relapse rates compared to the placebo, with a favorable safety profile [8, 11, 12].

Despite promising results from clinical trials, real-world evidence on the effectiveness and safety of ECU remains limited, particularly in diverse clinical populations with longer disease duration, comorbid conditions, or prior treatment failures. Additionally, there is insufficient data on the relationship between treatment duration and disability outcomes in real-life settings.

This study aims to evaluate the real-world effectiveness, safety, and predictors of clinical outcomes in individuals with AQP4-IgG-positive NMOSD treated with ECU.

## Methods

### Study population

This retrospective, multicenter study was conducted across 26 centers in five countries: Turkey, Argentina, Colombia, Montenegro, and Italy. The study population included 46 patients diagnosed with Aquaporin-4 antibody-positive (AQP4-IgG +) neuromyelitis optica spectrum disorder (NMOSD) who initiated ECU treatment between 2019 and 2025. Diagnosis was based on the 2015 International Consensus Diagnostic Criteria for NMOSD, with AQP4-IgG seropositivity confirmed exclusively using cell-based assay techniques. Exclusion criteria included seronegativity for AQP4-IgG or incomplete medical records. As ECU was administered as part of routine clinical care, no interventional procedures were applied specifically for this study. ECU was initiated in patients exhibiting ongoing disease activity despite adequate prior immunosuppressive therapy. Treatment-line classification followed national health policies, typically classifying azathioprine as first-line and rituximab as second-line therapy. Patients initially received azathioprine at a dose of 150 mg/day for at least 6 months. Those who experienced clinical relapses under azathioprine were escalated to rituximab-based therapy, administered as 1000 mg every 6 months, either as monotherapy or in combination with stable add-on treatment, including azathioprine (100 mg/day) or low-dose oral methylprednisolone (≤ 16 mg/day). Consequently, ECU was initiated in patients who continued to experience relapses despite at least one adequate course (minimum 6 months) of rituximab-based therapy. In a subset of patients who experienced relapses under rituximab monotherapy, treatment was switched to a combination of rituximab with azathioprine or low-dose methylprednisolone; patients who continued to relapse under this regimen were subsequently transitioned to ECU. ECU was administered according to a standard protocol: 900 mg once weekly for the first four doses, followed by 1200 mg every 2 weeks starting from the 5 week. All patients underwent continuous treatment accompanied by systematic monitoring of clinical outcomes, including relapse frequency, expanded disability status scale (EDSS) scores, and paraclinical parameters such as MRI findings and routine laboratory investigations. The study received approval from the Bursa Uludağ University Faculty of Medicine Ethics Committee (2025/1–6).

### Data collection

Data were extracted from electronic medical records and standardized case report forms completed by the treating physicians. Collected variables included demographic characteristics: age, sex, age at disease onset, disease duration, and presence of comorbid autoimmune conditions. Family history of NMOSD or other autoimmune disorders was also recorded.

Treatment-related variables included prior and add-on immunosuppressive agents (oral corticosteroids, azathioprine, rituximab, tocilizumab, others), number of ECU infusions, duration of treatment (in months) were recorded. Vaccination status for meningococcus and pneumococcus, as well as the use of prophylactic antibiotics, were also documented.

Clinical outcomes included relapse frequency before and after ECU initiation, annualized relapse rate (ARR), remission status, relapse frequency in the two years prior to treatment, and clinical relapses during follow-up. EDSS scores were collected at treatment initiation (baseline), 6 months, 12 months, 18 months, and 24 months after treatment initiation. Radiological data included the presence of T2-hyperintense lesions, LETM, short-segment transverse myelitis, optic nerve involvement, area postrema syndrome, and brainstem lesions. Information regarding infusion reactions, adverse events, their severity (CTCAE grade), treatment discontinuation, and mortality was also recorded.

### Statistical analysis

The normality of the data distribution was assessed using the Shapiro–Wilk test. Descriptive statistics were presented as mean ± standard deviation or median (minimum–maximum) for continuous variables, and as frequency and percentage for categorical variables. For comparisons between two independent groups, the Mann–Whitney *U* test was used for non-normally distributed data. Categorical variables were analyzed using the Pearson Chi-square test, Fisher’s Exact test, and the Fisher–Freeman–Halton test where appropriate. In cases where statistical significance was detected, the Bonferroni correction was applied for multiple comparisons. A *p*-value of < 0.05 was considered statistically significant. All statistical analyses were conducted using IBM SPSS Statistics for Windows, Version 29.0.2.0 (IBM Corp., Armonk, NY, USA).

## Results

### Descriptive characteristics of the study population

A total of 46 patients diagnosed with NMOSD were included in the study and included patient data from 26 centers across five countries (Turkey, Argentina, Colombia, Montenegro, and Italy). The country breakdown of the study cohort is as follows: 42 patients (91.3%) from Turkey, 2 patients (4.3%) from Argentina, 1 patient (2.2%) from Colombia, 1 patient (2.2%) from Montenegro, and 1 patient (2.2%) from Italy. The majority were female (*n* = 42, 91.3%). The mean age at disease onset was 36.8 ± 11.7 years, and the mean disease duration was 9.4 ± 5.9 years. Autoimmune comorbidities were present in 10 patients (21.7%).

Among the initial symptoms, optic neuritis was the most frequently reported (82.6%), followed by transverse myelitis (63%). Less common presentations included area postrema syndrome (8.7%), brainstem syndrome (6.5%), tonic spasms (4.3%), diencephalic syndrome, itching, and cerebral syndrome (each 2.2%).

**Fig. 1:**
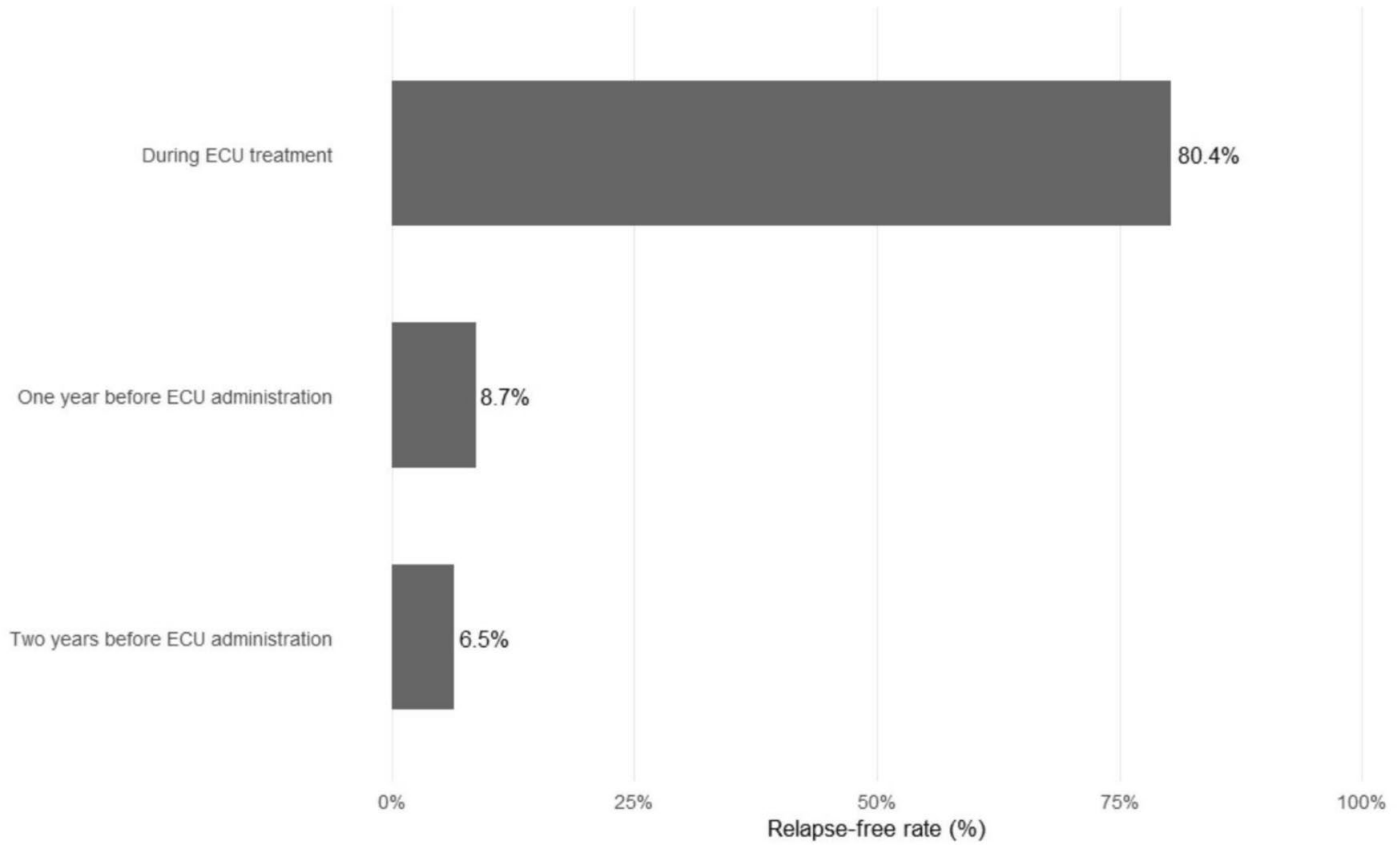
Percentage of relapse-free patients in the 2 years prior to eculizumab, 1 year prior, and during eculizumab treatment

The mean number of attacks before ECU initiation was 6.2 ± 3.7. The mean duration of prior immunosuppressive therapy was 37 ± 18 months. During the two years preceding ECU treatment, patients experienced a mean of 2.4 ± 1.6 attacks, and 1.4 ± 0.8 attacks during the final year before ECU initiation. After 2 years of ECU therapy, the mean relapse rate decreased to 0.3 ± 0.7. In our study cohort, the remission rate was 6.5% in the 2 years before ECU, 8.7% in the 1 year before treatment, and significantly increased to 80.4% during ECU therapy (Fig. 1). The ARR was calculated as 1.1 in the 2 years prior to treatment, 1.4 in the last year before treatment, and decreased to 0.1 under ECU therapy.

Regarding ECU treatment order, 1 patient (2.2%) received it as first-line therapy, 10 (21.7%) as second-line, 26 (56.5%) as third-line, and 9 (19.6%) as fourth-line. The single case of first-line ECU use represents an isolated, individualized therapeutic decision, likely based on a highly aggressive disease presentation that necessitated immediate, high-efficacy complement inhibition. Mean duration of ECU use was 27.2 ± 23.4 months, with a mean total dose number of 50.2 ± 44.5. Concomitant use of immunosuppressive agents (steroids or azathioprine) was observed in 13 patients (28.2%).

MRI findings prior to ECU therapy revealed optic nerve involvement in 50% of patients, area postrema involvement in 13.0%, LETM in 71.7%, short segment lesions in 13.0%, brainstem involvement in 2.2%, and cerebral involvement in 32.6%.

In terms of preventive measures, 97.8% had received meningococcal vaccination, and 76.1% had received pneumococcal vaccination before initiating ECU. Antibiotic prophylaxis was not administered. No infusion reactions were reported, while adverse events related to ECU were observed in seven patients (18.9%).

At the time of analysis, ECU treatment was ongoing in 40 patients (86.9%), while it had been discontinued in 6 patients (13.1%). New lesions on MRI during ECU treatment were noted in two patients (4.4%) (Table 1).

**Table 1:** Descriptive characteristics of the study population

Variable	NMOSD *n* = 46
Sex (female)	42 (91.3)
Age at disease onset (years)	36.8 ± 11.7
Disease duration (years)	9.4 ± 5.9
Autoimmune comorbidity present	10 (21.7)
Family history of NMO	1 (2.17)
Initial symptoms of the disease	
Optic neuritis	38 (82.6)
Transverse myelitis	29 (63.0)
Itching	1 (2.2)
Diencephalic syndrome	1 (2.2)
Area postrema syndrome	4 (8.7)
Tonic spasm	2 (4.3)
Brainstem syndrome	3 (6.5)
Cerebral syndrome	1 (2.2)
All attacks before the ECU	6.2 ± 3.7
Attacks during the past 2 years before ECU	2.4 ± 1.6
Attacks during 1 year before the ECU	1.4 ± 0.8
Attacks after 2 years of ECU treatment	0.3 ± 0.7
ECU usage order	
1 st line therapy	1 (2.2)
2nd line therapy (Prior drugs are RTX, or RTX + add-on therapy)	10 (21.7)
3rd line therapy (prior drugs are first Azathioprine, second RTX, or RTX-add-on)	26 (56.5)
4th line therapy (prior drugs are first Azathioprine, second RTX, or RTX-add-on, third Tocilizumab)	9 (19.6)
The mean duration of prior immunosuppressive therapy (months)	37 ± 18
Duration of ECU use/month	27.2 ± 23.4
Number of ECU doses	50.2 ± 44.5
Add-on therapy (steroid, azathioprine)	13 (28.2)
MRI findings before ECU	
Optic nerve involvement	23 (50)
Area postrema involvement	6 (13.0)
LETM	33 (71.7)
Short segment involvement	6 (13.0)
Brainstem involvement	1 (2.2)
Cerebral involvement	15 (32.6)
Vaccination status before ECU treatment	
Meningococcal vaccine	45 (97.8)
Pneumococcal vaccine	35 (76.1)
Antibiotic use before ECU treatment	0 (0)
Infusion reaction associated with ECU treatment	0 (0)
ECU-related adverse event	7 (18.9)
ECU status: ongoing/discontinued	40/6 (86.9)
New lesion on MRI during ECU treatment	2 (4.4)

Descriptive statistics were given as mean ± standard deviation, frequency, and percentage

### Longitudinal EDSS score changes

**Fig. 2:**
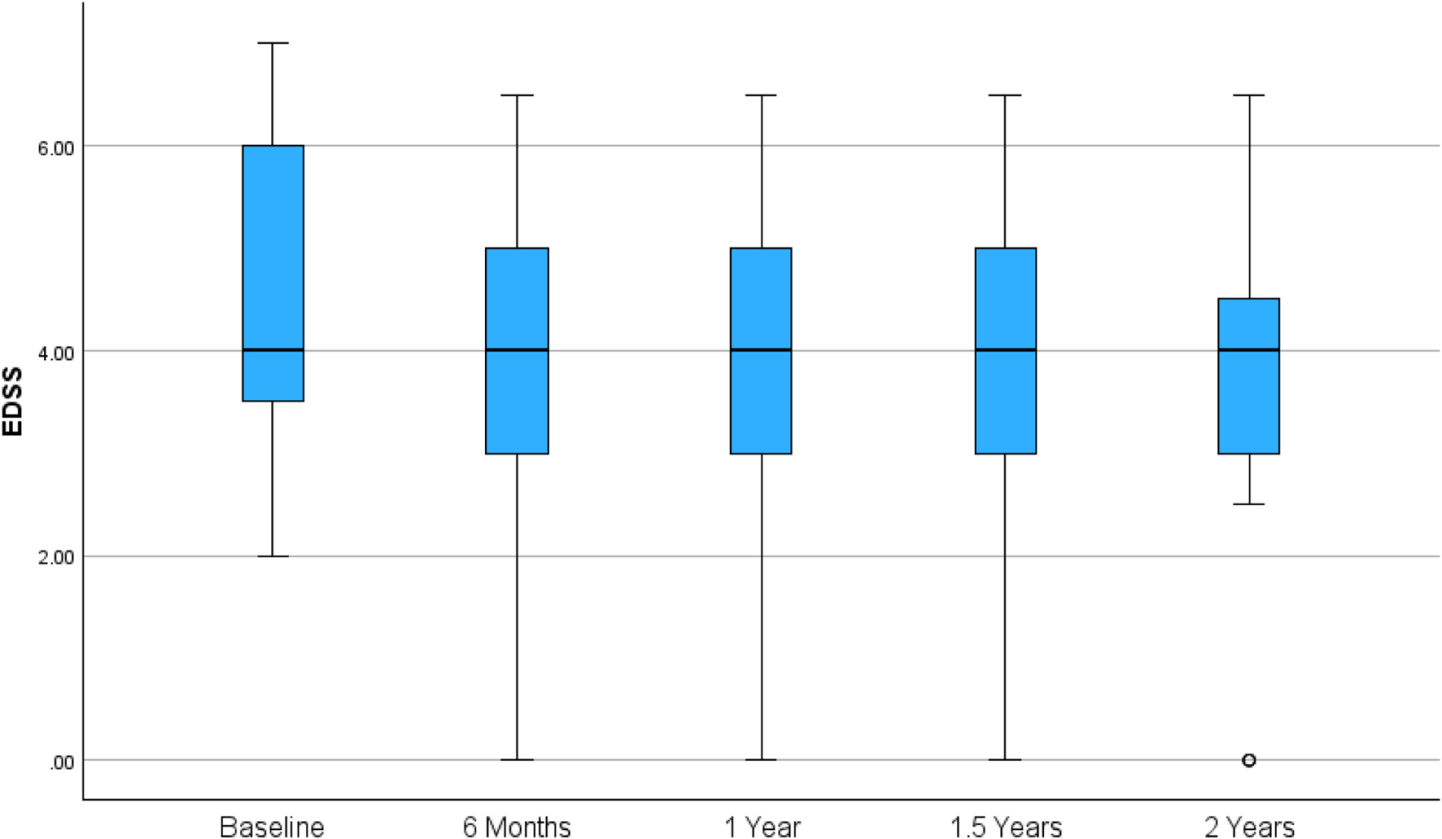
Mean EDSS scores over time (baseline, 6 months, 1 year, 1.5 years, 2 years) are shown with standard deviation bars

As shown in Fig. 2, mean EDSS scores showed overall improvement after the initiation of ECU therapy. At baseline, the mean EDSS was 4.2 ± 1.7, decreasing to 3.7 ± 1.8 at 6 months, 3.5 ± 1.7 at 12 months, and 3.4 ± 1.7 at 18 months. At the 24 month follow-up, a slight increase was observed (3.6 ± 1.8), although the value remained below baseline. This pattern suggests early clinical improvement for most patients, with a plateau or mild attenuation in a subset by the second year (Fig. 2).

### EDSS status categorization over time

**Fig. 3:**
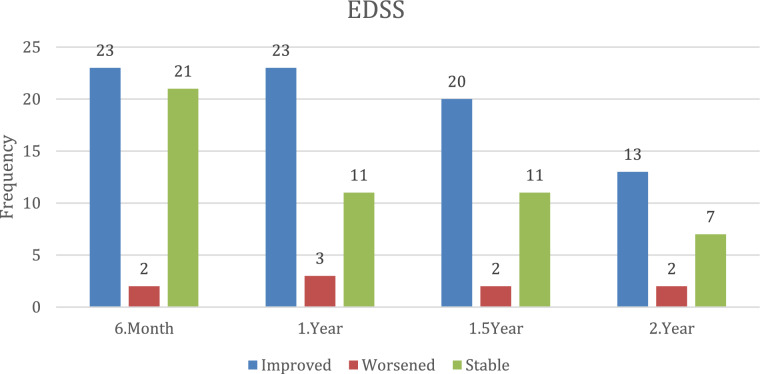
EDSS status change (improved, stable, worsened) over time (6 months, 1 year, 1.5 years, 2 years) with median edss scores shown as a line graph

Figure 3 illustrates the categorization of patients into three groups based on EDSS change at follow-up: improved, stable, and worsened. At 6 month follow-up, equal numbers of patients improved (*n* = 23) and remained stable/worsened (*n* = 23). The number of patients with improved EDSS remained unchanged at 12 months (*n* = 23), while the number with stable or worsened scores decreased to 14. Similar patterns were observed at 18 months (improved: *n* = 20; stable/worsened: *n* = 13) and at 24 months (improved: *n* = 13; stable/worsened: *n* = 9) (Fig. 3). These findings collectively indicate a beneficial effect of ECU on disability outcomes, particularly during the first year of treatment, with subsequent stabilization or sustained response in most patients.

### Comparison of clinical and radiological characteristics between patients with and without EDSS improvement at follow-up

To explore factors associated with EDSS improvement during ECU therapy, patients were stratified into 2 subgroups at each time point: those who demonstrated improvement and those who remained stable or worsened. At the 6 month follow-up, no significant differences were found in baseline characteristics between the two groups. However, there was a non-significant trend toward a higher frequency of multiple symptoms at disease onset in the non-improved group (*p* = 0.07).

At the 12 month and 18 month follow-ups, the presence of area postrema-type attacks prior to ECU treatment was significantly associated with EDSS improvement (*p* = 0.031 and *p* = 0.0327, respectively). Conversely, a spinal-type attack prior to treatment was significantly more common in the non-improved group at 18 months (*p* = 0.031), suggesting a possible association with poorer treatment response.

No statistically significant differences were found in other clinical features, including sex, age at onset, optic neuritis or transverse myelitis at onset, or baseline MRI findings. Use of add-on immunosuppressants (steroids or azathioprine) did not show a significant impact on EDSS outcomes, although a near-significant trend was observed for concomitant steroid use after 24 months (*p* = 0.05) (Table 2).

**Table 2:** Comparison of clinical and radiological characteristics between patients with and without EDSS ımprovement at each follow-up time

Variable	6 month			1 year			1.5 year			2 years		
	EDSS stabil/Worsened (*n* = 23)	Improved (*n* = 23)	*p*	EDSS stabil/Worsened (*n* = 14)	Improved (*n* = 23)	*p*	EDSS stabil/Worsened (*n* = 13)	Improved (*n* = 20)	*p*	EDSS stabile/ Worsened (*n* = 9)	Improved (*n* = 13)	*p*
Age at disease onset ≥ 40 years	8	7	0.753	6	6	0.470	5	6	0.714	3	5	1.000
Female sex	20	22	0.608	13	21	1.000	12	18	1.000	8	12	1.000
Optic neuritis at onset	20	18	0.699	12	17	0.683	11	15	0.676	8	10	0.616
Transverse myelitis at onset	17	12	0.127	10	15	1.000	9	14	1.000	6	9	1.000
Itching at onset	1	0	1.000	1	0	0.378	1	0	0.394	0	0	–
Diencephalic syndrome at onset	1	0	1.000	1	0	0.378	1	0	0.394	1	0	0.409
Area postrema syndrome at onset	0	4	0.109	0	3	0.275	0	3	0.261	0	1	1.000
Tonic spasm at onset	2	0	0.489	1	0	0.378	1	0	0.394	1	0	0.409
Brainstem syndrome at onset	2	1	1.000	2	0	0.137	2	0	0.148	2	0	0.156
Cerebral syndrome at onset	0	1	1.000	0	1	1.000	0	1	1.000	0	1	1.000
Multiple symptoms at onset	17	10	0.071	11	11	0.065	10	11	0.278	7	7	0.380
MRI cerebral involvement before ECU	9	6	0.345	6	6	0.470	5	6	0.714	3	4	1.000
MRI optic nerve involvement before ECU	12	11	0.768	8	8	0.183	8	6	0.073	6	4	0.192
MRI area postrema involvement before ECU	3	3	1.000	1	5	0.376	1	5	0.364	0	2	0.494
MRI LETM before ECU	15	18	0.326	9	18	0.454	9	15	1.000	6	12	0.264
MRI short-segment spinal lesion	5	1	0.187	3	2	0.346	2	3	1.000	1	1	1.000
Brainstem involvement on MRI	0	1	1.000	0	0	-	0	0	-	0	0	-
Area postrema type attack before ECU	2	6	0.243	0	7	0.031	0	7	0.027	0	4	0.115
Brainstem-type attack before ECU	4	0	0.109	2	1	0.544	2	0	0.148	2	0	0.156
Diencephalic-type attack before ECU	3	5	0.699	3	3	0.653	3	3	0.659	3	2	0.609
Optic neuritis attacks before ECU	20	15	0.084	13	15	0.112	12	14	0.202	9	9	0.115
Spinal type attack before ECU	21	16	0.135	14	16	0.031	13	15	0.131	9	10	0.240
Add-on steroid with ECU	2	6	0.243	1	6	0.217	1	5	0.364	0	5	0.054
Add-on AZT with ECU	2	3	1.000	0	4	0.276	0	4	0.136	0	3	0.240
Autoimmune comorbidity	6	4	0.475	3	6	1.000	3	5	1.000	1	1	1.000
Duration of ECU treatment	22 (5–40)	23 (5–120)	0.517	28.5 (16–40)	24 (12–120)	0.588	28 (16–40)	28.5 (17–120)	0.456	30 (24–40)	38 (24–120)	0.110
Number of ECU doses	44 (9–68)	42 (4–218)	0.517	47.5 (18–68)	45 (22–218)	0.936	46 (18–68)	57 (27–218)	0.404	63 (18–68)	69.5 (44–218)	0.095

## Discussion

In this multicenter study of seropositive NMOSD patients, ECU was associated with sustained clinical stabilization with low radiological activity during follow-up. The average EDSS scores remained stable or improved at 6, 12, 18, and 24 months. An improvement in relapse metrics was also observed compared with the pre-treatment period. Comparative analyses revealed phenotype-related signals for EDSS outcomes, with prior area postrema attacks associated with improvement and spinal attacks linked to poorer outcomes at specific time points.

Our study demonstrated significant improvements in both remission rates and ARR under ECU. Before therapy, the remission rate was below 10%, whereas during therapy, it increased to over 80%. These findings are consistent with the PREVENT trial, which reported that patients had an approximately 94% lower adjudicated relapse risk and an ARR of 0.02 with ECU, although no short-term effect on disability progression was observed [11]. Long-term data confirmed sustained efficacy over time [13]. Post-marketing surveillance in Japan similarly demonstrated minimal relapse activity (0.02 per patient-year) with a favorable safety profile [14]. In Europe, the real-world cohort from the neuromyelitis optica study group found that 88% of patients were attack-free with stable EDSS scores. However, older, more disabled patients had a greater frequency of serious infections and death [15]. Our younger cohort, with lower baseline disability, is consistent with the efficacy signal while providing complementary insights into safety.

We further examined phenotype-specific correlates of disability change within our cohort. Most significantly, our findings provide exploratory phenotype − specific signals regarding disability outcomes, a finding not previously addressed by the PREVENT study. The presence of area postrema–type attacks before ECU treatment was associated with a favorable prognosis and was significantly more frequent among patients who showed EDSS improvement at 12 and 18 months. Specifically, the number of patients with an AP-type attack who showed EDSS improvement was 7 at both 12 months (*p* = 0.031) and 18 months (*p* = 0.027), demonstrating a statistically significant association. In contrast, a history of spinal attacks was significantly associated with non-improvement at 18 months (*p* = 0.031). This contrasting phenotype signal suggests a differential tissue repair capacity influenced by complement inhibition, a novel and biologically plausible finding in a real-world setting, likely related to the reduced plasticity of long spinal cord tracts compared to brainstem nuclei [16, 17]. Axonal regeneration in the spinal cord is constrained by strong myelin-associated inhibitory signals [16] and limited intrinsic regenerative capacity [17], whereas brainstem structures may have greater potential for compensation through synaptic reorganization [18]. These mechanisms may underlie the adverse prognostic impact of spinal attacks in NMOSD [19].

Adverse events occurred in seven patients during ECU treatment. Reported events were: one anterior-thigh abscess, 1 soft-tissue tumor on the back, 2 cases of severe pneumonia, 1 acute kidney injury, 1 otitis, and 1 recurrent infection (pneumonia, upper respiratory tract infection, and pyelonephritis). In 5 of these patients, therapy was paused for approximately 1 month and then successfully reintroduced without further complications; in 2 patients, ECU was permanently discontinued due to adverse events. Overall, four patients discontinued therapy: 2 for adverse events, 1 for persistent relapses despite treatment, and 1 due to reimbursement issues.

This safety profile in routine practice aligns with findings from both controlled and observational studies. In the PREVENT trial, most adverse events were non-serious (e.g., respiratory infections, headache), although one death occurred from empyema [11]. In Japanese post-marketing surveillance, 26.8% of patients reported adverse events [14], and a European cohort identified a signal for serious infections, including meningococcal sepsis, with approximately 19% treatment discontinuation, mainly among older and more disabled patients [15]. Our experience with temporary interruption and subsequent successful re-challenge in 5 patients suggests that this may be a viable management strategy after adverse event resolution, provided that vaccination, prophylaxis, and infection surveillance are rigorously maintained. The absence of meningococcal infections in our cohort further supports the effectiveness of high vaccination coverage, consistent with Japanese real-world data [14]. It should be noted, however, that attributing causality is challenging in real-world settings, as add-on immunosuppression, comorbidities, and baseline disability likely contribute to infection risk.

Pregnancy outcomes under complement inhibition have emerged as an area of clinical concern. In our cohort, one patient became pregnant while receiving ECU therapy for AQP4-IgG-positive NMOSD. With informed consent and multidisciplinary monitoring, ECU treatment was continued. However, the pregnancy ended in spontaneous miscarriage at the 7th gestational week. To our knowledge, pregnancy outcomes under ECU in NMOSD are rarely reported. The pivotal PREVENT trial excluded pregnant individuals [11], and available real-world evidence is limited [20, 21]. Our case, therefore, adds to the scarce literature and highlights unresolved questions regarding the safety of complement inhibition during early gestation in this patient population. Evidence from other ECU-treated diseases provides complementary insights. In myasthenia gravis, case reports and small series have described favorable maternal and neonatal outcomes when ECU was continued during pregnancy, especially in refractory or life-threatening cases [22, 23]. In atypical hemolytic uremic syndrome, where the largest body of pregnancy data exists, studies have reported both successful pregnancies and miscarriages under ECU therapy, indicating that while the drug may often be used safely, individualized risk–benefit assessment remains essential [24–26]. Collectively, these data underscore the need for NMOSD-specific pregnancy registries and prospective studies to better guide clinical decision-making.

Aligned with these efficacy–safety considerations, therapeutic positioning should be framed within an individualized decision-making paradigm. Our data support effectiveness and a manageable safety profile in routine practice. Except for one patient, all individuals in our cohort had received at least one prior immunosuppressive therapy, and the majority were treated with ECU as monotherapy. Therefore, treatment selection should remain personalized, taking into account prior treatment history, relapse burden, comorbidities, and infection risk, in line with real-world evidence and ongoing prospective registry efforts such as ECUP4 in France [27].

From a methodological perspective, it is important to interpret these findings in the context of both strengths and limitations. The strengths include a multinational, practice-based sample and detailed EDSS and phenotype subgrouping. First, a limitation of the study is the lack of detailed calculations of annual relapse rates under each specific prior immunosuppressive treatment. While we report the mean duration and relapse numbers prior to ECU, the retrospective nature limited our ability to granularly analyze prior treatment efficacy per agent. Second, the study lacked a concurrently matched control group receiving standard immunosuppressive therapy. Instead, we relied on intra-patient comparisons of pre-treatment vs. on-treatment outcomes. While this supports effectiveness, the use of pre–post comparisons of annualized relapse rates may introduce bias, including regression to the mean, particularly given the fluctuating disease activity characteristic of NMOSD. Although ECU initiation was largely restricted to patients with documented treatment failure under maintenance therapies, residual confounding cannot be fully excluded. Third, despite a high incidence of optic neuritis as an initial symptom, standardized visual acuity outcome measures were not available for comprehensive retrospective assessment across all centers. While our findings support the effectiveness of ECU in a treatment-refractory real-world population and results regarding area postrema phenotypes and disability outcomes remain exploratory, larger prospective controlled studies with standardized outcome assessments are needed to robustly evaluate ECU’s efficacy relative to standard-of-care therapies.

In conclusion, our findings strengthen the evidence for complement inhibition as an effective treatment option while highlighting key clinical considerations for patient selection and monitoring. In this cohort, ECU was linked to strong relapse prevention and stabilization of EDSS, with only 7 patients experiencing adverse events and a total of four discontinuations. These results support ECU as a valuable therapy for AQP4-IgG–positive NMOSD, while stressing the need for careful safety monitoring and personalized treatment decisions. Special attention should be given to spinal-attack history as a potential negative predictor at 12 months and area postrema involvement as a possible positive predictor of EDSS improvement under ECU. Although our study emphasizes clinical outcomes, the significant relapse prevention and EDSS stabilization suggest that the long-term benefits of ECU in preventing irreversible disability and reducing the need for costly acute relapse treatments (hospitalizations, plasma exchange) could make it cost-effective. Formal pharmacoeconomic studies are needed to confirm this in various healthcare systems.
